# Hashimoto's encephalopathy presenting with neurocognitive symptoms: a case report

**DOI:** 10.1186/1752-1947-4-337

**Published:** 2010-10-25

**Authors:** Carlos Canelo-Aybar, David Loja-Oropeza, Jose Cuadra-Urteaga, Franco Romani-Romani

**Affiliations:** 1 Department of Medicine, Arzobispo Loayza Hospital, Lima, Peru

## Abstract

**Introduction:**

Hashimoto's encephalopathy is a neurological disorder of unknown cause associated with thyroid autoimmunity. The disease occurs primarily in the fifth decade of life and may present in two types - a sudden vasculitic type or a progressive subacute type associated to cognitive dysfunction, confusion and memory loss.

**Case presentation:**

We report the case of a 62-year-old Hispanic woman, previously healthy, who developed a subacute onset of declining upper brain function. Serologic studies demonstrated high levels of antithyroid antibodies. Electroencephalographic and magnetic resonance image findings were consistent with Hashimoto's encephalopathy.

**Conclusion:**

Hashimoto's encephalopathy is a diagnosis of exclusion. This unusual disorder is often under-recognized because of the multiple and protracted neurocognitive manifestations; therefore, it is important to be aware of the clinical manifestations to make a correct diagnosis.

## Introduction

Hashimoto's encephalopathy (HE) is an uncommon neurologic syndrome associated with Hashimoto's thyroiditis. It was initially described in 1966 [1], and it remains a controversial disorder. The cause of HE has been proposed to be autoimmune because of its association with other immunologic disorders (myasthenia gravis, glomerulonephritis, primary biliary cirrhosis, pernicious anemia and rheumatoid arthritis), female predominance, inflammatory findings in cerebrospinal fluid (CSF) and response to treatment with steroids [1,2]. Other authors suggest that HE may represent an autoimmune cerebral vasculitis resulting from either endothelial inflammation or immune complex deposition [1-3].

Clinical findings are variable and nonspecific. In this case report, we present the case of a patient with subacute onset of declining upper brain functions associated with Hashimoto's thyroiditis.

## Case presentation

Over a five-month period, a 62-year-old Hispanic woman who was previously healthy developed tremor in the right arm, enuresis, slowness in performing her daily activities, walking difficulties and trouble with getting dressed. Additionally, her relatives observed transient episodes of disorientation and inappropriate irritability.

Initially, the patient was admitted to another hospital, where she was found to have apraxia, dysphasia, attention deficit and amnesic episodes. She had no sensory or motor deficits.

**Figure 1:**
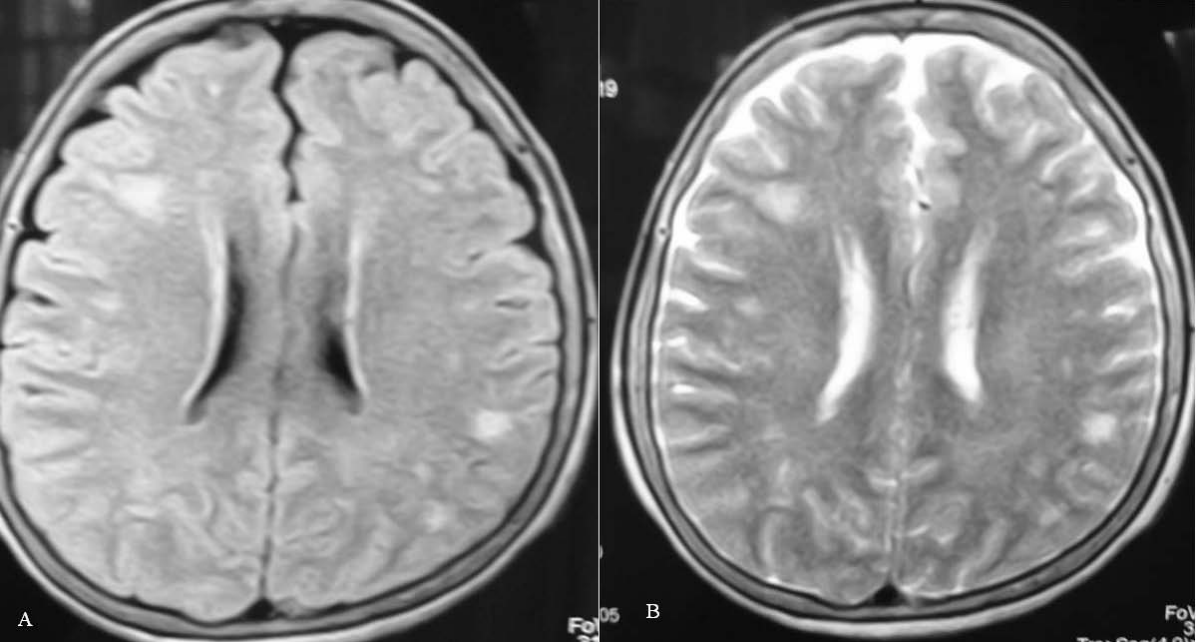
**Axial magnetic resonance images (MRI) of the brain demonstrating nodular subcortical lesions suggestive of demyelination in frontal and parietal lobes **. A) T1-weighted MRI. B) T2-weighted MRI.

Laboratory studies at that time revealed the presence of antithyroid antibodies as well as slightly high serum thyrotropin (TSH) concentration (Table 1). Examination of the CSF was normal. Magnetic resonance images (MRI) showed nodular focal subcortical lesions suggestive of demyelination (Figure 1). A diagnosis of encephalitis and hypothyroidism was made, and the patient received levothyroxine.

**Table 1:** Laboratory studies prior admission on Arzobispo Loayza Hospital

Studies	Value	Normal Range
Hemoglobin	11.7 g/dL	12-16 g/dL
Leukocyte count	3,000 cells/μL	4,500-10,000 cells/μL
Platelet count	800,000 cells/μL	150,000-400,000 cells/μL
INR*	1.7	1.0
Aspartate aminotransferase	39 U/L	0-37 U/L
Alanine aminotransferase	72 U/L	0-34 U/L
Albumin	3.7 g/dL	3.5-5.2 g/dL
Globulin	3.5 g/dL	2.5-3.0 g/dL
Thyroid-stimulating hormone	7.7 μU/mL	2.3-4.0 μU/mL
Free thyroxine (T4)	0.9 ng/mL	1.0-2.0 ng/dL
Antithyroglobulin antibody	135 IU/mL	<10.0 IU/mL
Antithyroid peroxidase	715 IU/mL	<10.0 IU/mL

*INR: International Normalizated Ratio of prothrombin time

Fifteen days later, the patient had two episodes of inappropriate behavior and transient anterograde amnesia. With these symptoms, she was admitted to our hospital.

The laboratory examination showed no significant change compared with the patient's previous laboratory results except normalization of hemogram values. Additionally, antinuclear antibody titer, anti-double-stranded DNA, anti-hepatitis B core antigen, hepatitis B surface antigen, anti-hepatitis C virus, lupic anticoagulant and Venereal Disease Research Laboratory test results were negative. Also, the anticardiolipin antibody IgG level was 10.8 U/GPL (reference range, <23 U/GPL), anticardiolipin antibody Ig M was 5.9 U/MPL (reference range, < 11 U/MPL), porphobilinogen deaminase level was 10.3 nmol/seg/L (reference range, 9.2-19.1 nmol/seg/L), 24-hour urine porphobilinogen was 1.22 mg/24 h (reference range, 0.2-2.00 mg/24 h), and 24-hour urine-delta-aminolevulinic acid level was 2.46 mg/24 h (reference range, 0.1-4.5 mg/24 h).

**Figure 2:**
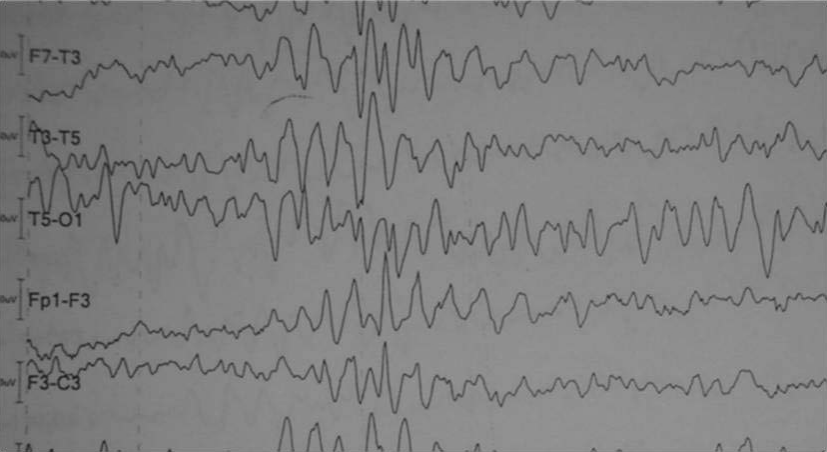
**An electroencephalogram showing a slowing background activity with theta waves and paroxysmal activity at hyperventilation maneuver **.

Considering the clinical and laboratory findings, a diagnosis of encephalopathy of undetermined origin was made. The electroencephalogram (EEG) showed a slow background activity with theta waves and paroxysmal activity at the hyperventilation maneuver (Figure 2). The thyroid biopsy showed lymphocytic chronic thyroiditis, and a diagnosis of HE was considered.

At discharge, the patient was treated with prednisone at doses of 1 mg/kg body weight. Thirty days later, she was experiencing a mild improvement in her symptoms. However, she never returned for her scheduled follow-up medical appointments.

## Discussion

HE is an unusual neurologic disorder whose etiology, pathogenesis and histologic characteristics are unclear. A systematic review published in 2003 [1] reported only 85 well-documented cases in the literature; however, this syndrome may be underrecognized. A hospital-based epidemiologic study of neurologic symptoms consistent with HE estimated its prevalence to be about 2.1 per 100,000 [4]. The disorder occurs more frequently between age 44 to 46 years, with a female-to-male ratio of four to one [1,5].

The clinical manifestations usually include acute to subacute onset of confusion with alteration of consciousness. Two major patterns of presentation were described: (1) 25% of patients follow a stroke-like pattern of multiple recurrent episodes of focal neurologic deficits with a variable degree of cognitive dysfunction and consciousness impairment [1,2], and (2) the remaining 75% present with a diffuse progressive pattern of slow cognitive decline with dementia, confusion and hallucinations [1,2]. These two clinical patterns may overlap over the course of the disease. In this case report, our patient's clinical manifestations are more consistent with the second form of presentation, which is more common.

Two-thirds of patients may experience focal or generalized tonic-clonic seizures, and 12% may present with status epilepticus. Also, myoclonus or tremor is seen in up to 38% of patients; hyperreflexia and other pyramidal tract signs in 85% of patients; and psychosis, visual hallucinations and paranoid delusions have been reported in 25% to 36% of patients [1,2,5].

The mechanism of HE does not appear to be related to the thyroid status, which can vary greatly in patients with HE. In two recent reviews, 23% to 35% of patients had subclinical hypothyroidism, 17% to 20% had hypothyroidism, 7% had hyperthyroidism and 18% to 45% were euthyroid [1,5]. The development of neurologic symptoms may occur up to three years before the onset of autoimmune thyroiditis [6].

The presence of elevated serums levels of antithyroid antibodies remains an essential characteristic of HE diagnosis, and suggest the presence of thyroid autoimmunity [1,5]. Although in some cases, the diagnosis is supported by the association with Hashimoto's thyroiditis, it is possible that some patients develop HE without a concomitant clinical thyroid disease because asymptomatic thyroid autoimmunity is frequent in these patients [1,5].

The pathogenic role of thyroid antibodies remains unknown, there is no evidence that any antithyroid antibody reacts with brain tissue or affects nerve function, and there is no clear correlation between the severity of the neurologic symptoms and the concentration of these antibodies [1,4].

Antithyroid antibodies have also been related to other autoimmune conditions such as myopathy, depression, bipolar disease and dementia, but the prevalence of these antibodies in the general population (ranging from 2%-20%) make it difficult to establish whether a real association exists [7].

Infrequently, the titers of antithyroid antibodies (TPOAb and TgAb) are measured in the CSF. In one case series, nine of 12 patients with encephalopathy and elevated serum antithyroid antibodies had elevated CSF autoantibody titers [4]. A systematic review found that 13% of published cases of HE reported antithyroid antibodies in the CSF [5]. However, the titers of antithyroid antibodies in the CSF do not correlate with the clinical stage of the disease, and the sensitivity and specificity of this finding remain unclear [4,5].

An autoantibody against the amino terminal end of the enzyme α-enolase, an antigen of the thyroid and the brain, has been identified as a potential biomarker of HE [5,8]. A study found serum autoantibody reactivity in five of six patients with HE compared with two of 17 patients with Hashimoto's thyroiditis but no HE and in none of 25 healthy control subjects [8]. This antigen is also found in endothelial cells, suggesting an autoimmune vasculitic mechanism; however, this has not been confirmed by neuroimaging techniques [5].

In some patients, C-reactive protein and the erythrocyte sedimentation rate are elevated [9], and in one series, mild elevation of liver enzymes was found in 12 of 20 patients [9)], which is concordant with the mild elevation observed in our patient.

Although the CSF analysis results were normal in our patient, a lymphocytic pleocytosis has been found in 14% of reported patients; in 4% of patients, it may contain more than 100 cells/mm^3^. An elevated protein concentration occurs in 78% of patients, and in 20% of patients, it may be greater than 100 mg/dL. The blood glucose concentration is usually normal [1,2].

Nonspecific EEG abnormalities are seen in 90% to 98% of patients, which is usually a nonspecific slow background activity. The same pattern was observed in our patient. Focal spikes or sharp waves and transient epileptic activity are less common [2,10].

In a review of 82 patients with HE, brain computed tomography or MRI showed abnormalities in 49% such as cerebral atrophy, focal cortical abnormality, diffuse subcortical abnormality and nonspecific subcortical focal white matter abnormality. The latter was observed in our patient as subcortical foci of demyelination [1].

The differential diagnosis of HE must consider any condition associated with delirium, rapidly progressive dementia, seizures or focal neurologic deficits [5]. Thus, the list of diseases that can be confused with HE is vast, including stroke or transient ischemic attack, cerebral vasculitis, carcinomatous meningitis, toxic metabolic encephalopathies, paraneoplastic syndromes, Creutzfeldt-Jakob disease, degenerative dementia and psychiatric diseases [1,5].

The long-term prognosis is variable, although a high percentage of patients respond to treatment; others could have a progressive or a relapsing course [1,5]. The symptoms usually improve with glucocorticoid therapy; however, it is not necessary because of treatment. A systematic review of 85 cases published of HE found clinical response in 98% of patients treated with glucocorticoids, 92% of patients treated with glucocorticoids and levothyroxine and 67% of patients treated with levothyroxine only [1].

Although our patient had a mild improvement of her symptoms, the long-term effect of the therapy could not be assessed because the patient did not return for her follow-up medical appointments.

## Conclusion

HE frequently presents with a myriad of neurocognitive symptoms and normal findings in several different examinations. This syndrome may go unrecognized for a long time; therefore, it should be kept in mind when evaluating a patient with cognitive dysfunction and high titers of antithyroid antibodies.

## Consent

Written consent was obtained from the patient for publication of the case report and any accompanying images. A copy of the written consent is available for review by the Editor-in-Chief of the journal.

## Competing interests

The authors declare that they have no competing interests.

## Authors' contributions

CCA contributed to patient care, drafting the manuscript and literature review. JCU contributed to interpretation of data and drafting of the manuscript. FRR contributed to data collection and literature search for the manuscript. DLO contributed to patient care, drafting the manuscript, revision and approval of the manuscript. All authors read and approved the final manuscript.
